# On inferring evolutionary stability in finite populations using infinite population models

**DOI:** 10.1007/s00285-021-01636-9

**Published:** 2021-07-31

**Authors:** Chai Molina, David J. D. Earn

**Affiliations:** 1 Department of Ecology and Evolutionary Biology, Princeton University, Princeton, NJ 08544 USA; 2 International Institute for Applied Systems Analysis, Laxenburg, 2361 Austria; 3 Department of Mathematics and Statistics, McMaster University, Hamilton, ON L8S 4K1 Canada

**Keywords:** Evolutionary game theory, Finite populations, Evolutionary stability, Selection process, 92D15, 91A22, 60J20

## Abstract

Models of evolution by natural selection often make the simplifying assumption that populations are infinitely large. In this infinite population limit, rare mutations that are selected against always go extinct, whereas in finite populations they can persist and even reach fixation. Nevertheless, for mutations of *arbitrarily small phenotypic effect*, it is widely believed that in *sufficiently large populations*, if selection opposes the invasion of rare mutants, then it also opposes their fixation. Here, we identify circumstances under which infinite-population models do or do not accurately predict evolutionary outcomes in large, finite populations. We show that there is no population size above which considering only invasion generally suffices: for any finite population size, there are situations in which selection opposes the invasion of mutations of arbitrarily small effect, but favours their fixation. This is not an unlikely limiting case; it can occur when fitness is a smooth function of the evolving trait, and when the selection process is biologically sensible. Nevertheless, there *are* circumstances under which opposition of invasion does imply opposition of fixation: in fact, for the $n$-player snowdrift game (a common model of cooperation) we identify sufficient conditions under which selection against rare mutants of small effect precludes their fixation—in sufficiently large populations—*for any selection process*. We also find conditions under which—no matter how large the population—the trait that fixes *depends on the selection process*, which is important because any particular selection process is only an approximation of reality.

## Introduction

Adaptive dynamics is a widely used and extremely successful framework for investigating the evolution of continuous traits by natural selection (Brännström et al. 2013). In this framework, it is classically assumed that the population is infinite and well-mixed, and that any single mutation has an infinitesimally small phenotypic effect. One of its significant contributions is a simple method for identifying ***locally evolutionarily stable strategies*** (local ESSs). If residents are playing a local ESS then rare mutants playing a distinct but sufficiently similar strategy cannot invade the population (Geritz et al. 1998; Brännström et al. 2013).

The simplicity of the notion of local ESS depends on the mathematically convenient simplifying assumption that the population is infinite. In a finite population of size *N*, selection can oppose the invasion of a mutant strategy, yet favour its fixation (i.e., the offspring of a single mutant will replace the resident population with probability greater than 1/*N*, the fixation probability for a neutral mutation arising in a single individual). Thus, Nowak et al. (2004) proposed a refinement of the classical definition of evolutionary stability, requiring in addition to selection opposing invasion, that selection also oppose fixation of the mutant strategy; such a strategy is said to be an ${\varvec{ESS}}_{{{\varvec{N}}}}$ to emphasize the finite population size. Importantly, fixation probabilities depend on the selection process—which formalizes how fitness differences give rise to changes in population-level frequencies of traits over time [e.g., Der et al. (2011), Molina and Earn (2018)]—and hence the selection process can also affect whether or not a strategy is an $\text {ESS}_{\mathrm{N}}$.

In contrast to the conditions for evolutionary stability in finite populations, the adaptive dynamics condition for evolutionary stability does not explicitly address the possible fixation of mutants. The reason is that in an infinite, well-mixed population, a strategy that cannot invade will not fix: the effect of finitely many mutants on the residents’ fitness is “infinitely diluted” and therefore negligible (mutants can affect the residents’ fitness only if they constitute a non-negligible proportion of the population, in which case there must be infinitely many mutants). Consequently, mutants that are selected against when rare die out before they can affect residents (Metz et al. 1996). Thus, the price of the convenience of assuming the population is infinite is that by excluding the possibility of fixation of mutants that are selected against when rare, one ignores the selection process, and disregards the possibility that frequency-dependent selection can undermine the evolutionary stability of a strategy.

Since infinite-population models are so widely used, but are inherently limited in their ability to account for the effects of the population size and the selection process on evolutionary outcomes, it is important to understand under what conditions the predictions of such infinite-population models apply to more realistic, finite populations. In particular, we are interested in whether or not, and when, infinite-population models correctly predict evolutionary outcomes in sufficiently large populations. To this end, we focus on evolutionary stability of strategies in populations in which individuals’ fitnesses are determined by their payoffs from an $n$-player snowdrift game (a common model of cooperation).

A natural first step is to ask whether or not evolutionary outcomes in infinite populations correctly predict those in sufficiently large finite populations when mutations have arbitrarily small effect. The evolutionary outcomes of interest are discrete (e.g., “stable” or “unstable”), so they cannot be approximated to some degree of accuracy; predictions will either be right or wrong. Consequently, because infinite-population models do not specify a selection process, they can predict evolutionary outcomes in finite populations only if the outcomes in finite populations are independent of the selection process. Thus we ask more precisely: for any given finite population size, if selection opposes the invasion of mutants playing a strategy sufficiently similar to the residents’, does it necessarily oppose their fixation? In Sects. 2 and 4, we show that the answer to this question is “*no*”: for any population size *N*, no matter how large, it is possible to construct well-behaved payoff functions (and a selection process) such that there is a singular strategy at which selection opposes the invasion but favours the fixation of mutations of arbitrarily small effect. Such a singular strategy is evolutionarily stable according to adaptive dynamics, but is not an $\text {ESS}_{\mathrm{N}}$.

It is arguably reasonable to suppose that if infinite-population models are, indeed, good approximations of *sufficiently large* finite populations, exactly *how large* is large enough might depend on the game through which fitnesses are determined. Thus, as a second step, we ask: given any specific game through which fitnesses are determined, if the population is sufficiently large and selection opposes the invasion of mutants that are sufficiently similar to residents, does it necessarily oppose their fixation? Sect. 5 addresses this question and shows that this is not true in general—even for a fixed game, the selection process is important, no matter how large the population. However, we identify a simple condition on $n$-player snowdrift games under which, if selection opposes the invasion of sufficiently similar mutants, it generically also opposes their fixation in sufficiently large populations, regardless of the selection process. When this condition holds, it provides a justification for analyzing evolutionary stability in finite populations using infinite-population models, and in particular, for ignoring the selection process in this context.

Importantly, there is no simple rule of thumb determining how large is “large enough”; this depends on the specifics of the selection process and the game that determines fitness. That is, for any finite population, no matter how large, an analysis based on a framework that ignores population size and the selection process is not sufficient in general to determine evolutionary outcomes.

## Model

Our analyses are presented in the context of a standard model for the evolution of cooperation, the $n$-player snowdrift game (Doebeli et al. 2004; Cornforth et al. 2012; Lion and van Baalen 2008; Killingback et al. 2010; Wakano and Iwasa 2012; Zhang et al. 2013; Chen et al. 2012; Zhong et al. 2008; Ito et al. 2015; Sasaki and Okada 2015; McNamara et al. 2008).

**Payoffs:** Members of a group of $n$ individuals make costly contributions to a public good, generating a benefit (available to all $n$ group members) that depends on the total contribution made by all group members. Denoting the cost of the focal individual’s contribution $x$ by $C(x)$ and the benefit to a focal individual in a group in which the total contribution is $\tau$ by $B(\tau )$, the focal individual’s payoff is

1 $$\begin{aligned} B(\tau )-C(x) . \end{aligned}$$

**Group formation:** Groups that play the snowdrift game are formed by sampling $n$ individuals uniformly and at random from the population without replacement. The population is thus well-mixed in the sense that individuals are equally likely to be sampled to play.

**Fitness:** Strategies are assumed to be inherited and therefore subject to selection. For simplicity, we assume that individuals play many rounds of the game between reproductive events (i.e., at each time step) and that their fitnesses are simply their average payoffs (1) from these games.

**Group and population sizes:** We assume that the group size $n\ge 2$. (If there were only one individual in a “group” then its optimal strategy would not depend on the behaviour of others and evolutionary game-theoretic considerations would be irrelevant.) The population can be infinite ($N=\infty$); if it is finite, we assume that it is larger than the group size, i.e., $N>n$; in particular, $N\ge 3$. (If the entire population were to play the game together, i.e., for $n=N$, individuals contributing the least would always have the highest fitness, so populations would inevitably evolve to defection, i.e., contributing nothing.)

**Trait substitution:** Mutations are assumed to be sufficiently rare that no more than two strategies are present in the population at any time.

## Strategy dynamics

### Infinite populations

When the snowdrift game described above is played in an infinite population ($N=\infty$), the evolution of strategies (i.e., contributions to the public good) is well-described by the canonical equation of adaptive dynamics (Dieckmann and Law 1996). A strategy $X$ is ***evolutionarily stable*** if (Geritz et al. 1998; Brännström et al. 2013)

1. it is ***singular***, i.e., directional selection vanishes in its vicinity (or more precisely, the local fitness gradient vanishes as it is approached),
  2a $$\begin{aligned} B'(nX)- C'(X)&= 0, \end{aligned}$$
  and
2. selection ***opposes invasion*** of mutants playing an arbitrarily similar strategy (which is ensured by requiring that the fitness of an invading mutant as a function of the mutant strategy is concave when mutants play the resident strategy)
  2b $$\begin{aligned} B''(nX)-C''(X)&<0 . \end{aligned}$$

### Finite populations

To find evolutionary outcomes of the $n$-player snowdrift game (Sect. 2) when played in a finite population ($N<\infty$), we use a framework for analyzing evolutionary stability in finite populations introduced in Molina (2016). We denote by $\delta \overline{W}_{\!\epsilon }(x,X)$ the ***difference in mean fitness*** of mutants (playing $x$) and residents (playing $X$), where $\epsilon$ is the proportion of the population playing the mutant strategy $x$ (so $\epsilon$ takes one of the values $0, \frac{1}{N}, \dots , \frac{N-1}{N}, 1$).

Similar to the infinite-population case, selection opposes invasion of a population of residents playing $X$ by mutants playing (an arbitrarily similar strategy) $x$ if the expected fitness of such mutants is lower than for residents, that is, $\delta \overline{W}_{\!\epsilon }(x,X)$ attains a local maximum as a function of $x$ for $x=X$. This occurs if the following two conditions hold.

1. The resident strategy $X$ is ***singular***, i.e., directional selection vanishes in its vicinity when mutants are rare, or more precisely, for $\epsilon =1/N$^1^, the local fitness difference gradient vanishes as it is approached, that is, $\left. \partial _x\delta \overline{W}_{\!\epsilon }(x,X)\right| _{x=X} = 0$; see Definition 4.3.5 in Molina (2016). For the snowdrift game, the condition for a singular strategy can be written
  3a $$\begin{aligned} \left( \frac{N-n}{N-1}\right) B'\big (nX\big )- C'(X)= 0 \end{aligned}$$
  [cf. Equation (4.64) in Molina (2016)].
2. When mutants are rare, the fitness of an invading mutant as a function of the mutant strategy is concave when mutants play the resident strategy, i.e., for $\epsilon =1/N$, $\left. \partial ^2_x\delta \overline{W}_{\!\epsilon }(x,X)\right| _{x=X} <0$. For the snowdrift game, this concavity condition can be written
  3b $$\begin{aligned} -C''(X) + \frac{N-n}{N-1} B''(nX)<0 \end{aligned}$$
  [cf. Equations (4.64) and (4.71) in Molina (2016)].

Equation (3a) is a necessary condition for selection opposing invasion of mutants playing strategies sufficiently similar to the residents: if (3a) does *not* hold, then the fitness difference between the invading mutant and residents is either increasing or decreasing as a function of the mutant’s strategy; in the increasing (resp. decreasing) case, invading mutants contributing slightly more (less) than the residents obtain higher fitness than the residents.^2^

In a finite population ($N<\infty$), the assumptions that define our model framework (Sect. 2) do not completely determine the strategy dynamics that unfold following the introduction of a mutant. In particular, fixation probabilities naturally depend on how fitnesses are used to determine changes in the frequencies of the two traits that are present in the population over time [e.g., the Moran or Wright–Fisher processes; see Moran (1962), Ewens (2012) and Nowak (2006)]. In Molina and Earn (2018), we formally define and analyze such ***selection processes***, which are Markov processes describing populations in which

1. there are at most two types of individuals (cf. the trait substitution assumption above);
2. there are no mutations;
3. the number of individuals of the type that has a higher mean fitness in a given generation is expected to increase in the next generation.

Without specifying a particular selection process, it is in general impossible to identify strategies that are $\text {ESS}_{\mathrm{N}}$s (i.e., evolutionarily stable in a population of size *N*); whether selection opposes the *fixation* of mutants playing a strategy sufficiently similar to the residents depends on the selection process.

In the next two subsections, we introduce further notation related to the mean fitness difference, and a class of selection processes that we will use in later sections.

#### Curvatures of the mean fitness difference

We now introduce convenient notation to simplify the Taylor expansion of the mean fitness difference $\delta \overline{W}_{\!\epsilon }(x,X)$ in the mutant strategy $x$ about a singular resident strategy $X$.

Using Eq. (3a) and the identity $\delta \overline{W}_{\!\epsilon }(X,X) = 0$ (neutral mutations do not confer a fitness advantage), for any number of mutants *i* ($1\le i\le N-1$), as well as the fact that the local fitness difference gradient $\left. \partial _x\delta \overline{W}_{\!\epsilon }(x,X)\right| _{x=X}$ is independent of the proportion of mutants, $\epsilon$ (see footnote 1 above) we can write the mean fitness difference

4 $$\begin{aligned} \delta \overline{W}_{\!\epsilon =i/N}(x,X) = \frac{1}{2}\omega _{i}\Delta x^2 + \mathcal {O}\!(\Delta x^3), \end{aligned}$$

where $$\Delta x= x-X$$, and

5 $$\begin{aligned} \omega _{i} :=\left. \partial ^2_x\delta \overline{W}_{\!\epsilon =i/N}(x,X)\right| _{x=X} ,\qquad i=1,\dots ,N-1, \end{aligned}$$

are the ***fitness difference curvatures***. Note that $\omega _{i}$ depends on the resident strategy $X$, but we make this dependence implicit for notational convenience.

Using Equations (4.64) and (4.71) of Molina (2016), the coefficient $\omega _{i}$ is given by

6 $$\begin{aligned} \omega _{i}&= \left( 1+ 2 (n-1)\frac{i-1}{N-1}-\frac{(n-1)\big [2(i-1)(n-2)+(N-2)\big ]}{(N-1)(N-2)}\right) B''(nX) - C''(X). \end{aligned}$$

In particular,

7 $$\begin{aligned} \omega _{1}&= -C''(X) + \frac{N-n}{N-1} B''(nX), \end{aligned}$$

so setting

8 $$\begin{aligned} \Delta \omega&:=2\frac{(n-1)(N-n)}{(N-1)(N-2)}B''(nX) , \end{aligned}$$

we have

9 $$\begin{aligned} \omega _{i} = \omega _{1} +(i-1)\Delta \omega . \end{aligned}$$

#### Symmetric birth-death processes

Some of our analysis involves identifying situations in which selection favours fixation. To that end, in Appendix A we define a class of biologically sensible selection processes—which we call ***symmetric birth-death (or SBD) processes***—for which fixation probabilities can be conveniently expressed in terms of differences in mean fitness. If there are *i* mutants in the population (with $1\le i\le N-1$), and if we denote the mean fitness difference by

10 $$\begin{aligned} \delta _i := \delta \overline{W}_{\!\epsilon = i/N}(x,X) , \end{aligned}$$

then the inverse of the fixation probability is

11 $$\begin{aligned} \frac{1}{p_{\mathrm{fix}}} = 1 + \sum _{j=1}^{N-1}\prod _{i=1}^{j}\left[ 1-\phi \,\delta _i + \mathcal {O}\!(\delta _i^2)\right] , \end{aligned}$$

where $\phi >0$ is a parameter that depends on which SBD process is chosen. SBD processes are used in Sect. 4 for analyses that depend only on Eq. (11) and in Sect. 6.2 for numerical simulations; the particulars of how SBD processes are defined (in Appendix A) are not essential to understand the results. In Sect. 5 and Sect. 6.3, we present more general results that are not specific to SBD processes.

## Selection can oppose invasion but favour fixation of arbitrarily similar mutants

In this section, we demonstrate that for any given population size *N* and any group size $n<N$, there are games for which selection opposes invasion but favours fixation (of mutant strategies that can be arbitrarily close to a singular strategy played by residents).

Consider the evolution of contributions to an $n$-player snowdrift game (as described in Sect. 2) in a finite population of size *N* governed by an SBD selection process (defined in Appendix A). In this situation, we show that it is possible to find benefit and cost functions, $B$ and $C$, and a resident strategy $X$, such that

mutants that play a strategy ($x$) that is different from—but sufficiently similar to—the resident strategy ($X$) obtain *lower fitness when rare*, yet *selection favours the fixation of such mutants*.

The conditions for this are stated precisely in Proposition 1, which we prove in Appendix B.

### Proposition 1

Consider an evolving population of finite size *N*, where fitnesses are determined by playing the $n$-player snowdrift game as described in Sect. 2. If residents play a singular strategy [i.e., a strategy *X* that satisfies Eq. (3a)], and in addition,

12 $$\begin{aligned} 0\;<\; B''(nX)\;<\; \frac{N-1}{N-n}C''(X) \;<\;\left( 1 + 2\frac{n- 1}{N} \right) B''(nX), \end{aligned}$$

then for any sufficiently similar strategies $x$ (i.e., for $|x-X|$ sufficiently small), selection opposes the invasion of mutants playing $x$, but favours their fixation under any SBD selection process (Sect. 3.2.2 and Appendix A).

It is easy to find functions *B* and *C* that satisfy the conditions in Proposition 1. In Sect. 6.2, we construct explicit examples of games that satisfy the hypotheses of Proposition 1 and therefore exhibit fixation of strategies that are opposed by selection when rare.

### Remark 2

Maynard Smith and Price’s original definition (1973) of evolutionary stability, stated in the context of 2-player games, listed two situations in which a resident strategy $X$ is evolutionarily stable against a mutant strategy $x$:

1. residents obtain a higher payoff than mutants when playing against a resident;
2. residents and mutants obtain the same payoff when playing against a resident, but residents obtain a higher payoff than mutants when playing against a mutant.

The first condition is motivated by situations in which a mutant will only ever encounter residents and residents will almost always encounter other residents; then a mutant that obtains a lower payoff (and consequently a lower fitness) than residents will almost surely die out. Implicitly, it is assumed that if selection opposes invasion, then it also opposes fixation. The second condition is relevant when selection does *not* oppose invasion, in which case the effect of mutants on the payoffs (and therefore fitnesses) of residents must be taken into account. Under this condition, while the initial mutant’s payoff is equal to the residents’, if there come to be more than one mutant, then their average payoff is less than that of residents due to the effect of interactions with other mutants.

Proposition 1 shows that in a finite population, the first of Maynard Smith and Price’s conditions is not sufficient to ensure evolutionary stability. This condition ensures only that selection opposes the mutant’s *invasion*, whereas frequency-dependent effects can still cause selection to favour the *fixation* of the mutant strategy.

## Evolutionary outcomes can depend on the selection process, even in large populations

In Sect. 4 we fixed the *population size* *N* and found conditions guaranteeing that selection opposes invasion but favours fixation; these conditions are satisfied by many snowdrift games. In this section, we fix the *game* (a snowdrift game with specific benefit and cost functions and a fixed group size^3^) but *not* the population size. We consider situations in which the game has an ESS if played in an infinite population and ask whether it also has an $\text {ESS}_{\mathrm{N}}$ if played in a sufficiently large finite population.

Proposition 3 below (proved in Appendix C) provides the answer, which is not as simple to state as one might hope. The existence of an infinite-population singular strategy $X^*_{\infty }$ generically implies the existence of a finite-population singular strategy $X^*_{N}$ when the same game is played in a finite population of sufficiently large size *N*; in fact, $X^*_{N}\rightarrow X^*_{\infty }$ as $N\rightarrow \infty$. We identify a condition [namely, condition (16)] guaranteeing that for sufficiently large population size *N*, $X^*_{N}$ is a (local) ***universal*** ${\varvec{\mathsf{{ ESS}}}}_{\varvec{\mathsf{{ N}}}}$ (U$\text {ESS}_{\mathrm{N}}$), that is, selection opposes the invasion and fixation of mutations of arbitrarily small effect, *regardless of the selection process*. However, if condition (16) does not hold, then the evolutionary stability of $X^*_{\infty }$ does not generally imply that $X^*_{N}$ is evolutionarily stable: we identify a condition under which, when residents play $X^*_{N}$, selection may either favour or oppose the fixation of mutations of arbitrarily small effect, *depending on the selection process*. In Sect. 6.3, we also construct explicit examples of snowdrift games that exhibit this behaviour.

### Proposition 3

In a snowdrift game as defined in Sect. 2, suppose the benefit and cost functions, $B$ and $C$, and the group size $n$, are such that there is a strategy $X^*_{\infty }>0$ satisfying the adaptive dynamics condition for evolutionary stability (2), and at which

13 $$\begin{aligned} nB''(nX^*_{\infty }) - C''(X^*_{\infty }) \ne 0 \end{aligned}$$

(which holds generically^4^). If this same game is played in a finite population of sufficiently large size *N*, then for each such *N* there is a singular strategy $X^*_{N}$, and

14 $$\begin{aligned} X^*_{N}\rightarrow X^*_{\infty }\quad \text {as}\quad N\rightarrow \infty . \end{aligned}$$

If, in addition, the fitness difference curvature $\omega _{N-1}$ [Eq. (5)] is negative for sufficiently large *N*, i.e., if there exists a population size $\underline{N}$ such that

15 $$\begin{aligned} N\ge \underline{N}\quad \implies \quad \omega _{N-1} < 0 , \end{aligned}$$

then there exists $\underline{N}^*\ge \underline{N}$ such that for any $N\ge \underline{N}^*$, $X^*_{N}$ is a U$\text {ESS}_{\mathrm{N}}$. A sufficient condition for such an $\underline{N}$ to exist is that

16 $$\begin{aligned} \lim _{N\rightarrow \infty }\omega _{N-1} <0. \end{aligned}$$

Conversely, if there exists $\overline{N}$ such that

17 $$\begin{aligned} N\ge \overline{N}\quad \implies \quad \omega _{N-1} > 0, \end{aligned}$$

then there exists ${\overline{N}}{}^*\ge {\overline{N}}$ such that for all $N\ge {\overline{N}}{}^*$, for mutations of arbitrarily small effect, selection favours fixation for some selection processes, but opposes fixation for other selection processes; a sufficient condition for such an $\overline{N}$ to exist is that

18 $$\begin{aligned} \lim _{N\rightarrow \infty }\omega _{N-1}> 0. \end{aligned}$$

Conditions (16) and (18) are easy to check because the limit can be expressed directly in terms of the benefit and cost functions: Eqs. (8) and (9) give

19 $$\begin{aligned} \omega _{N-1}=(2n-1)\frac{N-n}{N-1}B''\left( nX^*_{N}\right) - C''\left( X^*_{N}\right) . \end{aligned}$$

Since, in addition, $X^*_{N}\rightarrow X^*_{\infty }$ as $N\rightarrow \infty$ (14), we have

20 $$\begin{aligned} \lim _{N\rightarrow \infty }\omega _{N-1}= (2n-1)B''\left( nX^*_{\infty }\right) -C''\left( X^*_{\infty }\right) . \end{aligned}$$

Note that condition (15) [condition (17)] is more general than condition (16) [condition (18)]: the sign of $\lim _{N\rightarrow \infty }\omega _{N-1}$ being negative (positive) is not necessary for the existence of $\underline{N}$ $\left( \,\overline{N}\,\right)$, because it is possible that $\omega _{N-1}<0$ ($>0$) for all sufficiently large *N*, but that $\lim _{N\rightarrow \infty }\omega _{N-1}=0$. However, Eq. (20) implies that $\lim _{N\rightarrow \infty }\omega _{N-1}$ exists and vanishes iff $(2n-1)B''\left( nX^*_{\infty }\right) -C''\left( X^*_{\infty }\right) =0$, which generically is *not* satisfied. In other words, generically, either condition (16) or condition (18) holds.

In the unlikely situation that *neither* condition (15) *nor* (17) holds (which can only happen if $\lim _{N\rightarrow \infty }\omega _{N-1}=0$), there are two possibilities:

- There are increasing, unbounded sequences $\left\{ \overline{N}_i\right\} _{i\in \mathbb {N}}$ and $\{\underline{N}_{i}\}_{i\in \mathbb {N}}$ such that $\omega _{\underline{N}_{i}-1} < 0$ and $\omega _{\overline{N}_i-1} > 0$ for all $i\in \mathbb {N}$: in this case, corollary 5.4 and lemma 4.6 in Molina and Earn (2018) (respectively) imply that $X^*_{\underline{N}_{i}}$ is a U$\text {ESS}_{\mathrm{N}}$ and $X^*_{\overline{N}_i}$ is *not* a U$\text {ESS}_{\mathrm{N}}$ for all $i\in \mathbb {N}$. For any $N\in \{\overline{N}_i\}_{i\in \mathbb {N}}$, if residents play $X^*_{N}$, some selection processes favour fixation of mutations of arbitrarily small effect, while other selection processes oppose their fixation.
- The fitness difference curvature $\omega _{N-1}$ vanishes for all sufficiently large *N* (i.e., there exists $N_0$ such that $\omega _{N-1}=0$ for all $N>N_0$): in this case, it is possible that $x=X^*_{N}$ is a minimum, maximum or inflection point of $\delta \overline{W}_{\!(N-1)/N}\left( x,X^*_{N}\right)$ for all sufficiently large *N*. Consequently, if $\omega _{1}<0$ it is still possible that $X^*_{N}$ is a U$\text {ESS}_{\mathrm{N}}$; but, it is also possible that some selection processes favour fixation of mutations of arbitrarily small effect, while other selection processes oppose their fixation.

## Examples

In this section, we illustrate the predictions of Propositions 1 and 3 with examples, using a subclass of snowdrift games that we define in Sect. 6.1. The particular examples are then described in Sects. 6.2 and 6.3.

### A class of quadratic snowdrift games

Consider a snowdrift game (Sect. 2) with quadratic benefit and cost functions [similar to Doebeli et al. (2004)],

21a $$\begin{aligned} B_{\nu , \xi }(\tau )&= b_{2}\tau ^2 + b_{1}\tau , \end{aligned}$$

21b $$\begin{aligned} C_{\nu , \xi }(x)&= c_{2}x^2+ c_{1}x, \end{aligned}$$

where the coefficients are

22a $$\begin{aligned} b_{1}&= 0, \end{aligned}$$

22b $$\begin{aligned} b_{2}&=1,\end{aligned}$$

22c $$\begin{aligned} c_{1}&= 2,\end{aligned}$$

22d $$\begin{aligned} c_{2}&= \frac{\xi -n}{\xi -1}\bigg (1+ \nu \frac{n-1}{\xi }\bigg ), \qquad \xi>n, \quad 2>\nu \ge 1. \end{aligned}$$

We denote such a game for particular $\nu$ and $\xi$ as $\mathcal {G}_{n}\left( \nu , \xi \right)$, and the family of all such games for fixed group size $n$ as23Note that games in this class differ only in their cost functions.

#### Singular strategies

When snowdrift games with quadratic benefit and cost functions [Eq. (21)] are played in an infinite population, solving Eq. (2a) gives the unique singular strategy

24a $$\begin{aligned} X^*_{\infty }= \frac{c_{1} - b_{1}}{2\left( nb_{2} - c_{2}\right) }, \end{aligned}$$

whereas when they are played in a finite population of size $N>n$, Eq. (3a) gives the singular strategy,

24b $$\begin{aligned} X^*_{N}= \frac{c_{1} - \frac{N-n}{N-1}b_{1}}{2\left( \frac{N-n}{N-1}nb_{2} - c_{2}\right) }. \end{aligned}$$

Note, however, that for some choices of the coefficients ($b_{1}$, $b_{2}$, $c_{1}$, $c_{2}$), $X^*_{\infty }$ and $X^*_{N}$ can be negative (and therefore biologically irrelevant). For the specific benefit and cost coefficients given by Eq. (22), i.e., for all games in the class $\mathcal {F}_{n}$, Eqs. (24a) and (24b) become

25a $$\begin{aligned} X^*_{\infty }&= 1\bigg /\left[ n- \frac{\xi -n}{\xi -1}\bigg (1+ \nu \frac{n-1}{\xi }\bigg )\right] , \end{aligned}$$

25b $$\begin{aligned} X^*_{N}&= 1\bigg /\left[ \frac{N-n}{N-1}n- \frac{\xi -n}{\xi -1}\bigg (1+ \nu \frac{n-1}{\xi }\bigg )\right] , \end{aligned}$$

It is straightforward to show that $X^*_{N}>X^*_{\infty }>0$ for any $\xi >n$ and $\nu \in [1,2)$.

#### Sufficient condition for evolutionary stability in an infinite population

To guarantee that the singular strategy $X^*_{\infty }$ given by Eq. (25a) is evolutionarily stable when played in an infinite population, a sufficient condition is that

26 $$\begin{aligned} \xi>2n\qquad \text {and} \qquad \nu > \frac{\xi }{\xi -n}. \end{aligned}$$

To see this, we verify that condition (26) implies that condition (2b) is satisfied: With quadratic benefit and cost functions, condition (2b) yields $c_{2} > b_{2}$, and inserting Eq. (22) gives

27 $$\begin{aligned} \frac{\xi -n}{\xi -1}\bigg (1+ \nu \frac{n-1}{\xi }\bigg ) > 1, \end{aligned}$$

which simplifies to

28 $$\begin{aligned} (\xi -n)\nu&> \xi . \end{aligned}$$

This is equivalent to condition (26) if $\xi >2n$.

### Evolutionary games with different outcomes in finite populations and infinite populations

Given a finite population size *N*, we now consider the subclass of games $\mathcal {G}_{n}\left( \nu , \xi \right)$ for which $\xi =N$, i.e., $\{\mathcal {G}_{n}\left( N,\nu \right) \in \mathcal {F}_{n}\vert 2>\nu \ge 1\}$. Although $\mathcal {G}_{n}\left( N,\nu \right)$ is parameterized using the given *N*, note that the games in this subclass can also be played in an infinite population.

**Fig. 1:**
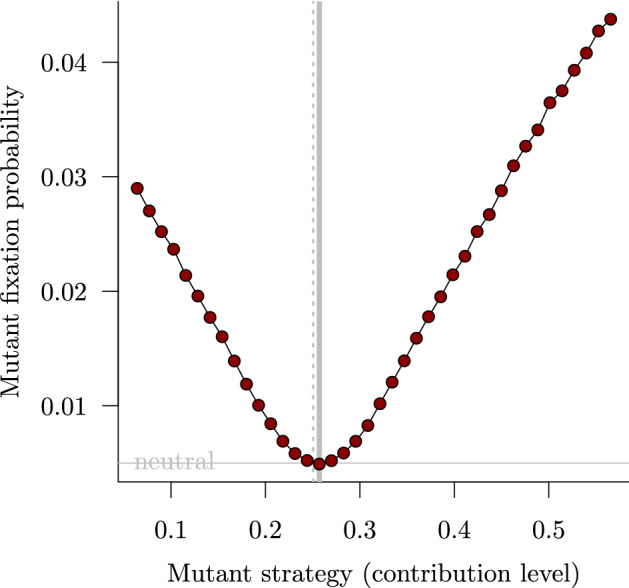
Selection opposing invasion but favouring fixation in a quadratic snowdrift game (Sect. 6.1; $\nu =3/2$, $\xi =200$) with group size $n=5$ and total population size $N=200$. The finite-population singular strategy ($X^*_{N}=0.2571$) is shown with a thick vertical grey line. The associated infinite-population ESS ($X^*_{\infty }=0.2506$) is shown with a thin dashed vertical grey line. The fixation probability of a neutral mutation ($1/N=0.005$) is shown with a horizontal grey line. The red dots show the fixation probability of mutants when residents play the $\text {ESS}_{\mathrm{N}}$, based on $10^7$ simulations for each mutant strategy, under a symmetric birth-death (SBD) selection process with transition probability ratio $R(\delta _i) = e^{-\delta _i}$ (so $\phi =1$ in Eq. (11); see Eq. (41) in Appendix A) (color figure online)

When a game in $\mathcal {G}_{n}\left( N,\nu \right)$ is played in a finite population of size *N*, Proposition 1 applies. Thus, under an SBD selection process (Appendix A), if residents play the singular strategy $X^*_{N}$, selection favours fixation of mutations of arbitrarily small effect, so $X^*_{N}$ is not an $\text {ESS}_{\mathrm{N}}$. Since cooperative strategies ($X>0$) that are not singular cannot be $\text {ESS}_{\mathrm{N}}$s, a game $\mathcal {G}_{n}\left( N,\nu \right)$ does not have a cooperative $\text {ESS}_{\mathrm{N}}$ when played in a population of size *N* under any SBD process. By contrast, if the same game $\mathcal {G}_{n}\left( N,\nu \right)$ is played in an infinite population, then $X^*_{\infty }$ [given below in Eq. (29a)] is an ESS for any $\xi =N>2n$ (see Sect. 6.1.2). We corroborate the prediction of evolutionary instability for finite *N* using individual-based simulations in Fig. 1.

To verify that Proposition 1 holds for any game $\mathcal {G}_{n}\left( N,\nu \right)$ (with $2>\nu \ge 1$), note first that when $\xi =N$, Eq. (25a) reduces to

29a $$\begin{aligned} X^*_{\infty }&= 1\bigg /\left[ (n-1)\frac{N-n}{N-1} \left( \frac{N}{N-n} - \frac{\nu }{N}\right) \right] >0, \end{aligned}$$

and Eq. (25b) becomes

29b $$\begin{aligned} X^*_{N}&= 1\bigg /\left[ (n-1)\frac{N-n}{N-1} \left( 1- \frac{\nu }{N}\right) \right] , \end{aligned}$$

which is positive for any $N\ge 2$ because $2> \nu >0$. Next, substituting Eq. (21) into condition (12) gives

30 $$\begin{aligned} 0< b_{2}< \frac{N-1}{N-n}c_{2} <\left( 1 + 2\frac{n- 1}{N} \right) b_{2}. \end{aligned}$$

For the specific coefficients of the benefit and cost functions given by Eq. (22), condition (30) becomes

31 $$\begin{aligned} 0<1< \frac{N-1}{N-n}\Big (\frac{N-n}{N-1}\Big )\bigg (1+ \nu \frac{n-1}{N}\bigg ) <\left( 1 + 2\frac{n- 1}{N} \right) , \end{aligned}$$

which manifestly holds for $\nu \in [1,2)$.

### Games for which evolutionary outcomes differ between infinite and arbitrarily large but finite populations

We now apply Proposition 3 to identify games in the class $\mathcal {F}_{n}$ that have an ESS when played in an infinite population but—depending on the selection process—either have, or do not have, an $\text {ESS}_{\mathrm{N}}$ when played in arbitrarily large finite populations.^5^

To do this, we must find games $\mathcal {G}_{n}\left( \nu , \xi \right) \in \mathcal {F}_{n}$ that (i) have an infinite population ESS $X^*_{\infty }$, (ii) satisfy condition (13), and (iii) have the property that there is a population size $\overline{N}$ such that condition (17) is satisfied.

First, to ensure that there is always an infinite population ESS, we assume $\nu$ and $\xi$ satisfy condition (26).

Second, we note that for games in $\mathcal {F}_{n}$, condition (13) simplifies to

32 $$\begin{aligned} \xi ^2+\nu \xi + n\nu&\ne 0, \end{aligned}$$

which holds because $\nu$, $\xi$ and $n$ are all positive. Hence condition (32) [and therefore condition (13)] holds for any $\nu \in [1,2)$.

Finally, we find that with $\overline{N}:=\lceil \xi \rceil$, condition (17) is satisfied: Inserting Eq. (22) in Eq. (19), we have

33 $$\begin{aligned} \omega _{N-1}= (2n-1)\frac{N-n}{N-1}- \frac{\xi -n}{\xi -1}\bigg [1+ \nu \frac{n-1}{\xi }\bigg ]. \end{aligned}$$

Substituting $\xi =N$ in Eq. (33) gives34which is positive because $N\ge 2$ and $2>\nu >0$. Since $(N-n)/(N-1)$ increases with *N*, so does $\omega _{N-1}$, and hence35

## Conclusion

Evolutionary game theory has been developed primarily under the approximation of an infinite background population (Lehmann 2012; Geritz et al. 1998; Doebeli et al. 2004; Zheng et al. 2007; Brown and Vincent 2008; Brännström et al. 2010; Deng and Chu 2011). In this setting, the notion of evolutionary stability can be formalized simply as “selection opposes invasion” and the details of the selection process are irrelevant. In finite populations, evolutionary stability requires the additional condition that “selection opposes fixation”, and which strategies are stable [$\text {ESS}_{\mathrm{N}}$s; see Nowak et al. (2004)] depends, in general, on the selection process (Molina and Earn 2018).

The traditional justification for the infinite population approximation is that sufficiently large finite populations behave as if they were infinite (Metz et al. 1996, Sect. 2.1). Here, we have challenged this conventional wisdom by demonstrating two mechanisms by which inferences drawn from evolutionary games played in an infinite population can turn out to be incorrect for more realistic, finite populations. First, we have shown that for any finite population size, there are biologically sensible situations in which selection favours the fixation of mutants, even though selection opposes their invasion (Proposition 1; example in Sect. 6.2). Second, we have identified conditions on $n$-player snowdrift games such that an infinite-population cooperative ESS exists, yet in a finite population—no matter how large—the existence of a cooperative $\text {ESS}_{\mathrm{N}}$ depends on the selection process, i.e., a cooperative $\text {ESS}_{\mathrm{N}}$ exists under some selection processes but not others (Proposition 3; example in Sect. 6.3).

Thus, while the infinite population approximation is convenient mathematically and leads to simpler predictions, those predictions can be misleading in finite populations (no matter how large). Infinite-population models should, therefore, be applied carefully and cautiously. To that end, we have also identified conditions under which the infinite population approximation *does correctly predict* evolutionary outcomes in sufficiently large finite populations. More precisely, Proposition 3 specifies conditions—on $n$-player snowdrift games—that guarantee that if an infinite-population ESS exists then in sufficiently large (but finite) populations there is a strategy that is *universally* evolutionarily stable (i.e., is an $\text {ESS}_{\mathrm{N}}$ for any selection process). Under these conditions, the adaptive dynamics framework is useful and correctly predicts evolutionary outcomes in sufficiently large (finite) populations.

## Appendices

### A Symmetric birth-death selection processes

Let $M_\mathrm{p}(t)$ be the number of mutant individuals at time *t*; since individuals are either mutants or residents, $M_\mathrm{p}(t)$ completely specifies the ***population state*** at time *t*. If $M_\mathrm{p}(t)=i$ for $1\le i\le N-1$ then both mutants and residents are present so we refer to a ***mixed population state***.

We define a discrete-time birth-death process that—based on the fitness difference between mutants and residents—changes the composition of the population over time; for convenience, we use $P$ to denote both this process, and the transition matrix that defines it. Specifically, we set

36a $$\begin{aligned} \MakeUppercase {P}_{{i},{j}}&=0,&\quad 0\le j<i-1 \quad \text {or}\quad i+1<j\le N, \end{aligned}$$

36b $$\begin{aligned} \frac{\MakeUppercase {P}_{{i},{i-1}}}{\MakeUppercase {P}_{{i},{i+1}}}&= R_i\left( \delta \overline{W}_{\!\epsilon = i/N}(x,X)\right) ,&1\le i\le N-1, \end{aligned}$$

36c $$\begin{aligned} \MakeUppercase {P}_{{i},{i}}&=1-\MakeUppercase {P}_{{i},{i+1}}-\MakeUppercase {P}_{{i},{i-1}},&1 \le i \le N-1, \end{aligned}$$

36d $$\begin{aligned} \MakeUppercase {P}_{{0},{0}}&=\MakeUppercase {P}_{{N},{N}}=1, \end{aligned}$$

where the dependence of the ***transition probability ratios*** $R_i$ ($i=1,\dots ,N-1$) on the mean fitness differences $\delta \overline{W}_{\!\epsilon }(x,X)$ (Sect. 3.2) will be specified shortly. The following two conditions must be satisfied for Eq. (36b) to make biological and mathematical sense:

- The right hand side of Eq. (36b) must be non-negative for any fitness difference $\delta \overline{W}_{\!\epsilon = i/N}(x,X)$ (otherwise some transition probabilities will be negative). Moreover, if $R_i\left( \delta \overline{W}_{\!\epsilon = i/N}(x,X)\right) =0$ for some *i* then mutants cannot go extinct (fixation of the mutants is certain) so we assume $R_i(\cdot )>0$. [The consistency condition (b) stated stated next also independently excludes the possibility that $R_i\left( \delta \overline{W}_{\!\epsilon = i/N}(x,X)\right) =0$.]
- The transition probability ratios $R_i$ must satisfy a consistency condition. If a population consists of *i* and $N-i$ individuals of types *A* and *B*, respectively, then the ratio of the probabilities that the number of individuals of type *A* increases, and decreases, must be independent of whether type *A* is labelled as the mutant or resident. Mathematically, if mutants and residents are interchanged, state *i* becomes state $N-i$, and the mean fitness difference $\delta \overline{W}_{\!\epsilon = i/N}(x,X)$ becomes $-\delta \overline{W}_{\!\epsilon = (N-i)/N}(x,X)$. Thus, writing $\delta _i=\delta \overline{W}_{\!\epsilon = i/N}(x,X)$ as in Eq. (10), we require that
  37 $$\begin{aligned} R_i(\delta _i)= \frac{1}{R_{N-i}(-\delta _i)}. \end{aligned}$$
- The transition probability ratios $R_i$ must be decreasing functions of the fitness difference. This assumption is motivated by the biologically sensible intuition that if the mutants have a fitness advantage over the residents, increasing this advantage should increase the probability that the number of mutants increases (and lower the probability that the number of mutants decreases). Conversely, if the residents have a fitness advantage over the mutants (i.e., $\delta \overline{W}_{\!\epsilon = i/N}(x,X)<0$) then increasing this advantage should *decrease* the probability that the number of mutants increases.

For simplicity, we assume that the ratios of probabilities of mutants increasing and decreasing ($\MakeUppercase {P}_{{i},{i-1}}/\MakeUppercase {P}_{{i},{i+1}}$) depend on the mean fitness difference $\delta \overline{W}_{\!\epsilon = i/N}(x,X)$, but not on the population state *i*, so that, with minor abuse of notation, only one transition probability ratio function is needed, $R_i=R$ for all $i=1,\dots ,N-1$, and

38 $$\begin{aligned} \frac{\MakeUppercase {P}_{{i},{i-1}}}{\MakeUppercase {P}_{{i},{i+1}}} = R\left( \delta \overline{W}_{\!\epsilon = i/N}(x,X)\right) , \quad 0<i<N. \end{aligned}$$

Condition (37) then becomes $R(\delta _i)R(-\delta _i)=1$. Since $R>0$, this is equivalent to

39 $$\begin{aligned} \log R(\delta _i)=-\log R(-\delta _i), \end{aligned}$$

so $r(\delta _i):=\log R(\delta _i)$ is an odd function. It follows that the transition probability ratio must be of the form

40 $$\begin{aligned} R(\delta _i)= e^{r(\delta _i)}, \end{aligned}$$

where $r:\mathbb {R}\rightarrow \mathbb {R}$ is odd. Since $R$ is decreasing, $r$ must also be decreasing.

We assume henceforth that $r$ is analytic in a neighbourhood of $\delta _i=0$. Because $r$ is odd, $r^{(n)}(0)=0$ for any non-negative even integer *n*, and letting $\phi =-r'(0)>0$, we have

41 $$\begin{aligned} r(\delta _i)=-\phi \delta _i+ \mathcal {O}\!(\delta _i^3). \end{aligned}$$

Consequently, for $i=1,\dots ,N-1$, Eq. (36b) gives

42 $$\begin{aligned} \frac{\MakeUppercase {P}_{{i},{i-1}}}{\MakeUppercase {P}_{{i},{i+1}}} = e^{r\left( \delta _i\right) }=1-\phi \delta _i+ \mathcal {O}\!(\delta _i^2). \end{aligned}$$

Equation (36) leaves some freedom in that we do not specify how likely it is for the population to remain at the same state (i.e., that $M_\mathrm{p}(t+1) = M_\mathrm{p}(t)$); this affects the speed of evolution, but not the fixation probabilities. For concreteness, we can set $\MakeUppercase {P}_{{i},{i}} = 0$ for all $i=1,\dots ,N-1$, in which case Eqs. (36b) and (36c) can be solved explicitly to obtain

43a $$\begin{aligned} \MakeUppercase {P}_{{i},{i+1}}&= \frac{1}{1+ R\left( \delta _i\right) }, \end{aligned}$$

43b $$\begin{aligned} \MakeUppercase {P}_{{i},{i-1}}&= \frac{R\left( \delta _i\right) }{1+ R\left( \delta _i\right) }. \end{aligned}$$

We have thus constructed a class of birth-death processes, determined by the choice of the logarithm $r$ of the transition probability ratio $R$, which must be a decreasing odd function (taking $r(\delta _i)=-\delta _i$ yields the simplest such process). These processes $P$ are well-behaved in the sense that for any resident and mutant strategies:

(I) at any time *t* the number of individuals of the type with higher fitness is expected to increase in the next time step,
(II) no new mutations are introduced, so that monomorphic populations are absorbing states of $P$, and
(III) starting from any mixed population state it is possible for the mutation to either fixate or become extinct, that is, the probabilities of these outcomes happening at some future time are nonzero.

Consequently, *P* is indeed a selection process as defined in Molina and Earn (2018). Moreover, $P$ satisfies the consistency condition (37) by construction and, for smooth $r$, depends smoothly on the mean fitness difference $\delta \overline{W}_{\!\epsilon = i/N}(x,X)$.

If initially one mutant individual playing $x$ enters the population ($M_\mathrm{p}(0)=1$), the probability that the mutation fixes can be calculated exactly (because *P* is a birth-death process) and is given by

44 $$\begin{aligned} p_{\mathrm{fix}}= \frac{1}{1 + \sum _{k=1}^{N-1}\prod _{j=1}^{k}\frac{\MakeUppercase {P}_{{j},{j-1}}}{\MakeUppercase {P}_{{j},{j+1}}}} \end{aligned}$$

[see, for example, Appendix C of Molina and Earn (2018) for a detailed proof]. Inserting Eq. (42), we obtain Eq. (11).

## B Proof of Proposition 1

Let $X$ be a singular strategy [Eq. (3a)] for which condition (3b) holds, so that selection opposes invasion of a population of residents playing $X$ by mutants playing $x$ sufficiently close to $X$. We aim to find additional conditions on the benefit and cost functions ($B$ and $C$) such that, for SBD processes (Sect. 3.2.2), selection *favours* the fixation of $x$ sufficiently near $X$, even though it opposes invasion of $x$ when rare. Denoting the fixation probability of the mutant strategy when one mutant individual initially enters the population by $p_{\mathrm{fix}}$ we wish to find conditions under which $p_{\mathrm{fix}}>1/N$.

First, observe that from Eq. (4), if $\omega _{i}<0$, then $\delta \overline{W}_{\!\epsilon =i/N}\left( x,X\right) <0$ for all $x$ sufficiently close to but different from $X$, and conversely, if $\omega _{i}>0$, then $\delta \overline{W}_{\!\epsilon =i/N}\left( x,X\right) >0$ for such mutants. Thus, if $\omega _{i}<0$ for all $i=1,\dots ,N-1$, then $\delta \overline{W}_{\!\epsilon =i/N}\left( x,X\right) <0$ for all $x$ sufficiently close to but different from $X$, so Corollary 5.4 in Molina and Earn (2018) implies that selection opposes fixation of such mutants regardless of the selection process. Thus, in order for selection to favour the fixation of mutants playing $x$ close to $X$, there must be some number of mutants *i* ($1\le i\le N-1$) for which $\omega _{i}>0$.

The definition of the fitness difference curvatures $\omega _{i}$ [Eq. (5)] implies that condition (3b) is equivalent to $\omega _{1}<0$. Therefore, to stack the odds in favour of the mutants fixing, we will require

45 $$\begin{aligned} \omega _{i}>0\quad \text {for all} \quad i=2,\dots ,N-1. \end{aligned}$$

Because $\omega _{i}$ is linear in *i* [Eq. (9)], this is achieved if

46 $$\begin{aligned} 0<-\omega _{1} < \Delta \omega . \end{aligned}$$

Using Eqs. (7) and (8), this is equivalent to

47 $$\begin{aligned} \frac{N-n}{N-1} B''(nX)< C''(X)&<\left( \frac{N-n}{N-1} + 2\frac{(n-1)(N-n)}{(N-1)(N-2)}\right) B''(nX)\nonumber \\&= \frac{N+ 2n-4}{N-2}\cdot \frac{N-n}{N-1}B''(nX). \end{aligned}$$

Henceforth, we assume that condition (46) [or equivalently, condition (47)] holds. Then, $\Delta \omega >0$, so the curvatures $\omega _{i}$ increase with *i*. Thus, we can bound fixation probabilities for invading mutants under an SBD process (Sect. 3.2.2) by substituting Eq. (4) into Eq. (11) to get

48 $$\begin{aligned} \frac{1}{p_{\mathrm{fix}}}&=1 + \sum _{k=1}^{N-1}\prod _{j=1}^{k}\Big (1- \frac{1}{2}\phi \,\omega _{j}\Delta x^2 + \mathcal {O}\!(\Delta x^3)\Big )\nonumber \\&\le 1 + \Big (1- \frac{1}{2}\phi \,\omega _{1}\Delta x^2 + \mathcal {O}\!(\Delta x^3)\Big )\left[ 1+ \sum _{k=2}^{N-1}\prod _{j=2}^{k}\Big (1-\frac{1}{2} \phi \,\omega _{2}\Delta x^2 + \mathcal {O}\!(\Delta x^3)\Big )\right] , \end{aligned}$$

where we have used $\phi >0$ [Eq. (41)] and $0<\omega _{2}\le \omega _{3}\le \cdots \le \omega _{N-1}$ to obtain inequality (48). Simplifying the term in square brackets gives

49 $$\begin{aligned} 1+ \sum _{k=2}^{N-1}&\prod _{j=2}^{k}\Big (1- \frac{1}{2} \phi \,\omega _{2}\Delta x^2 + \mathcal {O}\!(\Delta x^3)\Big ) \nonumber \\&=1+ \sum _{k=2}^{N-1}\Big (1- \frac{1}{2}\phi \,\omega _{2}\Delta x^2 + \mathcal {O}\!(\Delta x^3)\Big )^{k-1}\nonumber \\&= \sum _{k=0}^{N-2}\Big (1- \frac{1}{2}\phi \,\omega _{2}\Delta x^2 + \mathcal {O}\!(\Delta x^3)\Big )^{k}\nonumber \\&= \sum _{k=0}^{N-2} \sum ^k_{j=0} {{k}\atopwithdelims (){j}} \Big (- \frac{1}{2}\phi \,\omega _{2}\Delta x^2 +\mathcal {O}\!(\Delta x^3)\Big )^{j}\nonumber \\&= \sum _{k=0}^{N-2}\bigg [1 + k\Big (- \frac{1}{2}\phi \,\omega _{2}\Delta x^2 +\mathcal {O}\!(\Delta x^3)\Big ) + \mathcal {O}\!(\Delta x^4) \bigg ] \nonumber \\&= N-1 -\frac{(N-1)(N-2)}{2} \frac{1}{2}\phi \,\omega _{2}\Delta x^2 +\mathcal {O}\!(\Delta x^3), \end{aligned}$$

and hence

50 $$\begin{aligned} \frac{1}{p_{\mathrm{fix}}}&\le 1 + \Big (1- \frac{1}{2}\phi \,\omega _{1}\Delta x^2 + \mathcal {O}\!(\Delta x^3)\Big )\left( N-1 -\frac{(N-1)(N-2)}{2} \frac{1}{2}\phi \,\omega _{2}\Delta x^2 +\mathcal {O}\!(\Delta x^3)\right) \nonumber \\&=N -(N-1) \frac{1}{2}\phi \left( \omega _{1} +\frac{N-2}{2}\omega _{2} \right) \Delta x^2 + \mathcal {O}\!(\Delta x^3). \end{aligned}$$

It follows that if $\Delta x$ is sufficiently small then $\frac{1}{p_{\mathrm{fix}}}<N$ (so $p_{\mathrm{fix}}>\frac{1}{N}$ as desired) provided that the coefficient of $\Delta x^2$ in Eq. (50) is negative, i.e., provided that

51 $$\begin{aligned} 0&<\omega _{1} +\frac{N-2}{2} \omega _{2}\nonumber \\&=\omega _{1} + \frac{N-2}{2}\left( \omega _{1} + \Delta \omega \right) = \frac{N}{2}\omega _{1} +\frac{N-2}{2} \Delta \omega \nonumber \\&=\frac{N}{2}\left( -C''(X) + \frac{N-n}{N-1} B''(nX) \right) + \frac{N-2}{2}\left( 2\frac{(n-1)(N-n)}{(N-1)(N-2)}B''(nX)\right) \nonumber \\&=\frac{N}{2}\left( -C''(X) + \frac{N-n}{N-1} B''(nX) \right) + \frac{(n-1)(N-n)}{N-1}B''(nX)\nonumber \\&=\frac{N}{2}\left[ -C''(X) + \frac{N + 2(n- 1)}{N}\cdot \frac{N-n}{N-1} B''(nX)\right] . \end{aligned}$$

Thus, $$p_{\mathrm{fix}}>\frac{1}{N}$$ if

52 $$\begin{aligned} C''(X) <\frac{N + 2(n- 1)}{N}\frac{N-n}{N-1} B''(nX). \end{aligned}$$

Now recall that in order to ensure that selection opposes invasion of similar mutants, but that the mean fitness of mutants is higher than that of residents when the population contains two or more mutants, condition (47) must hold. Therefore, all that remains is to determine under what circumstances conditions (47) and (52) both hold. Because $N>n\ge 2$, we have

53 $$\begin{aligned} \frac{N + 2(n- 1)}{N} < \frac{N + 2n- 4}{N-2}. \end{aligned}$$

Furthermore, condition (47) also implies

54 $$\begin{aligned} B''(nX)>0, \end{aligned}$$

so we have

55 $$\begin{aligned} \frac{N + 2(n- 1)}{N} \cdot \frac{N-n}{N-1} B''(nX)<\frac{N + 2n- 4}{N-2} \cdot \frac{N-n}{N-1} B''(nX), \end{aligned}$$

that is, the right hand side of Eq. (52) is smaller than that of condition (47). Hence, both conditions (47) and (52) are satisfied if

56 $$\begin{aligned} B''(nX)< \frac{N-1}{N-n}C''(X) <\left( 1 + 2\frac{n- 1}{N} \right) B''(nX), \end{aligned}$$

in which case both $\omega _{1}<0$ and $p_{\mathrm{fix}}>\frac{1}{N}$, as desired.^6^$\square$

## Proof of Proposition 3

It can be shown that if the population size *N* is sufficiently large, there is a finite-population singular strategy $X^*_{N}$ [i.e., a solution of Eq. (3a)], and that the sequence of these singular strategies approaches $X^*_{\infty }$ as $N\rightarrow \infty$ (see Lemma 4 in Appendix D). Henceforth, assume without loss of generality that *N* is sufficiently large that the singular strategy $X^*_{N}$ exists. Taking the limit $N\rightarrow \infty$ in Eq. (7) we find

57 $$\begin{aligned} \lim _{N\rightarrow \infty }\omega _{1}=B''\left( nX^*_{\infty }\right) - C''\left( X^*_{\infty }\right) , \end{aligned}$$

which is negative because we assume that Eq. (2) is satisfied. Consequently, for *N* large enough, $\omega _{1}<0$.

Now, suppose that $\omega _{N-1}<0$ for all $N>\underline{N}$. Then, there exists $\underline{N}^*\ge \underline{N}$ such that if $N\ge \underline{N}^*$ then both $\omega _{1}$ and $\omega _{N-1}$ are negative. Since $\omega _{i}$ is linear in *i* [Eq. (9)], this implies that $\omega _{i}<0$ for all $i=1,\dots ,N-1$. Hence, from Eq. (4), we have $\delta \overline{W}_{\!\epsilon }(x,X^*_{N})<0$ for $x$ sufficiently close to but different from $X^*_{N}$. In other words, if residents play $X^*_{N}$, mutants playing a strategy that is sufficiently similar to the residents’ obtain a lower fitness than residents, regardless of the number of mutants (i.e., for all $i=1,\dots ,N-1$). Thus, selection opposes both invasion and fixation of such mutants regardless of the selection process (Molina and Earn 2018, Corollary 5.4), so $X^*_{N}$ is a U$\text {ESS}_{\mathrm{N}}$.

Similarly, if $\omega _{N-1}>0$ for all $N>\overline{N}$, then there exists ${\overline{N}}{}^*\ge \overline{N}$ such that $\omega _{1}<0$ and $\omega _{N-1}>0$, so $\omega _{i}$ changes sign as a function of *i*. Consequently, for all $N\ge {\overline{N}}{}^*$, when mutants play strategies that are arbitrarily similar to the residents, the mean fitness difference $\delta \overline{W}_{\!i/N}$ is positive for some numbers of mutants and negative for others. Consequently, from Lemma 4.6 in Molina and Earn (2018), it follows that selection favours fixation of such mutants for some selection processes, and selection opposes their fixation for other selection processes. $\square$

## D Existence and convergence of finite-population singular strategies

Lemma 4 below shows that the existence of a singular strategy when a game is played in an infinite population generically implies the existence of a corresponding singular strategy when it is played in a sufficiently large finite population.

### Lemma 4

(Existence of singular strategies in sufficiently large populations) Consider an evolving population of finite size *N* in which fitness is determined by payoffs from an $n$-player snowdrift game (Sect. 2) for which the second derivatives of the benefit ($B$) and cost ($C$) functions exist. Suppose that a singular strategy $X^*_{\infty }$ exists when the same snowdrift game is played in an infinite population, and that $nB''(nX^*_{\infty }) - C''(X^*_{\infty })\ne 0$ (which holds generically). Then, if the population size *N* is sufficiently large, a corresponding singular strategy $X^*_{N}$ exists. Furthermore, the sequence of finite-population singular strategies $X^*_{N}$ that result from playing this game in finite populations of different (sufficiently large) size approaches $X^*_{\infty }$ as $N\rightarrow \infty$.

### Proof

Letting

58 $$\begin{aligned} f(X,\rho ) :=\rho B'(nX) - C'(X), \end{aligned}$$

we have $f(X^*_{\infty },1)=0$, because $X^*_{\infty }$ is singular in an infinite population, and so satisfies Eq. (2a). Noting that $\partial _{X}f(X,\rho )\vert _{(X,\rho )=(X^*_{\infty },1)} = nB''(nX^*_{\infty }) - C''(X^*_{\infty })$, the hypothesis that $nB''(nX^*_{\infty }) - C''(X^*_{\infty })\ne 0$ implies that

59 $$\begin{aligned} \partial _{X}f(X,\rho )\vert _{(X,\rho )=(X^*_{\infty },1)} \ne 0. \end{aligned}$$

Therefore, from the implicit function theorem (e.g., Thomson et al. 2008, Theorem 12.40), there is a differentiable function $X(\rho )$ defined in a neighbourhood of $\rho =1$, such that

60 $$\begin{aligned} f\big (X(\rho ),\rho \big ) = \rho B'\big (nX(\rho )\big ) - C'\big (X(\rho )\big )=0. \end{aligned}$$

Now define

61 $$\begin{aligned} \rho _N:=(N-n)/(N-1) . \end{aligned}$$

Since $\rho _N\rightarrow 1$, it follows that for sufficiently large population size *N*, $f\big (X(\rho _N),\!\rho \big )\!=0$ can be solved implicitly to yield $X^*_{N}=X(\rho _N)$. These solutions $X^*_{N}$ are singular [i.e., solve Eq. (3a)]. Moreover, $X^*_{N}\xrightarrow {N\rightarrow \infty }X^*_{\infty }$ because $X(\rho )$ is continuous.

## Footnotes

1 Note that as shown in Molina (2016) (p. 138), in well-mixed populations, the local fitness difference gradient $\left. \partial _x\delta \overline{W}_{\!\epsilon }(x,X)\right| _{x=X}$ is independent of the proportion of mutants, $\epsilon$.

2 Moreover, because $\left. \partial _x\delta \overline{W}_{\!\epsilon }(x,X)\right| _{x=X}$ is independent of the proportion of mutants in the population, mutants that obtain a higher fitness when rare do so regardless of their frequency in the population, so by Corollary 5.4 of Molina and Earn (2018), selection favours their fixation.

3 By a fixed group size we mean that the size of the interacting group ($n$) is independent of the population size (*N*). For example, the typical size of groups travelling in cars that are obstructed by snowdrifts would be the same in small and large cities.

4 In particular, in the biologically sensible case of accelerating costs and decelerating benefits, condition (13) always holds.

5 For sufficiently large populations, condition (12) does not hold for quadratic snowdrift games, so Proposition 1 does not apply to these games. For such games, the second derivatives of the benefit and cost functions are independent of the resident strategy. Consequently, as the population size *N* is increased in condition (12), the rightmost expression approaches the second from the left expression (which is independent of *N*). The second from the right expression increases with *N*, so condition (12) must be violated for *N* large enough. Thus, we cannot say whether or not an $\text {ESS}_{\mathrm{N}}$ exists when such games are played in arbitrarily large but finite populations under SBD selection processes.
